# On Disciplinary Fragmentation and Scientific Progress

**DOI:** 10.1371/journal.pone.0118747

**Published:** 2015-03-19

**Authors:** Stefano Balietti, Michael Mäs, Dirk Helbing

**Affiliations:** 1 Professorship of Computational Social Science, ETH Zurich, Switzerland; Université de Montréal, CANADA

## Abstract

*Why are some scientific disciplines, such as sociology and psychology, more fragmented into conflicting schools of thought than other fields, such as physics and biology?* Furthermore, *why does high fragmentation tend to coincide with limited scientific progress?* We analyzed a formal model where scientists seek to identify the correct answer to a research question. Each scientist is influenced by three forces: (i) signals received from the correct answer to the question; (ii) peer influence; and (iii) noise. We observed the emergence of different macroscopic patterns of collective exploration, and studied how the three forces affect the degree to which disciplines fall apart into divergent fragments, or so-called “schools of thought”. We conducted two simulation experiments where we tested (A) whether the three forces foster or hamper progress, and (B) whether disciplinary fragmentation causally affects scientific progress and vice versa. We found that fragmentation critically limits scientific progress. Strikingly, there is no effect in the opposite causal direction. What is more, our results shows that at the heart of the mechanisms driving scientific progress we find (i) social interactions, and (ii) peer disagreement. In fact, fragmentation is increased and progress limited if the simulated scientists are open to influence only by peers with very similar views, or when within-school diversity is lost. Finally, disciplines where the scientists received strong signals from the correct answer were less fragmented and experienced faster progress. We discuss model’s implications for the design of social institutions fostering interdisciplinarity and participation in science.

## Introduction

*“Physical theories are like fixed points, towards which we are attracted. Our starting points may be culturally determined, our paths may be affected by personal philosophies, but the fixed point is there nonetheless.” [1]*

*“[…] our entire universe from the mythical Big Bang via the emergence of hydrogen and helium, galaxies, fixed stars, planetary systems, viruses, bacteria, fleas, dogs to the Glorious Arrival of Western Man is an artifact constructed by generations of scientist-artisans from a partly yielding, partly resisting material of unknown properties.” [2]*

The two quotes represent two opposing conceptions of science. The first quote by Steven Weinberg, Nobel Prize laureate in physics in 1979, represents a pure form of “scientific realism”, the conviction that science is able to accurately describe the world as it really is. Although the majority of natural scientists will agree with Weinberg’s statement, many scholars from the social sciences and humanities will disagree, as the second quote illustrates. This view implies that the process of developing scientific insights is largely influenced by social institutions, social norms, and conventions, making scientific knowledge the result of social construction [3–5].

Interestingly, the fundamental difference in philosophical views between social and natural scientists seems to reflect how their fields are socially structured and how they progress. On the one hand, the natural sciences have typically experienced a sequence of unification processes that have produced a generally accepted canon of theories. In physics, for instance, Newton unified the celestial and terrestrial forces, and Maxwell unified electricity and magnetism in one single force called electromagnetism. Later on, Einstein unified the Newtonian theory of gravitation and his special relativity with the theory of general relativity. In more recent years on a subatomic level, the strong force, and the weak force have been unified with the electromagnetic force in one single model. This model is called the Standard Model and was discovered, among others, by Steven Weinberg. In addition, the natural sciences are often characterized by a high rate of scientific progress in terms of explanatory power and descriptive accuracy of their theories.

The social sciences and humanities, on the other hand, are characterized by a high degree of fragmentation and slower scientific progress [5–8]. In most disciplines, one can identify fragmented communities or “schools” that base their work on competing theories and sometimes fundamentally different methods. In fact, with the sole exception of economics, all social sciences disciplines are still missing a core theory that is acknowledged by the majority of the researchers [9, 10]. In sociology, N. Mouzelis [8] describes the situation of its own field as a “[degeneration] into anarchy and cacophony, a total lack of communication between warring schools” (p. 149). In psychology, L. Cronbach uses the formulation “the two disciplines of psychology” to depict the long-standing contrast between experimentalist and correlational or “ethnic” psychologists [11]. This contrast is still so strong that often data generated within one tradition is disregarded by the other [12]. Quantitative evidence of the methodological schism is also found at the level of bibliographic coupling of scientific documents: psychology shows significant lower level of consensus, and much more fragmentation than physics [13].

**Fig 1:**
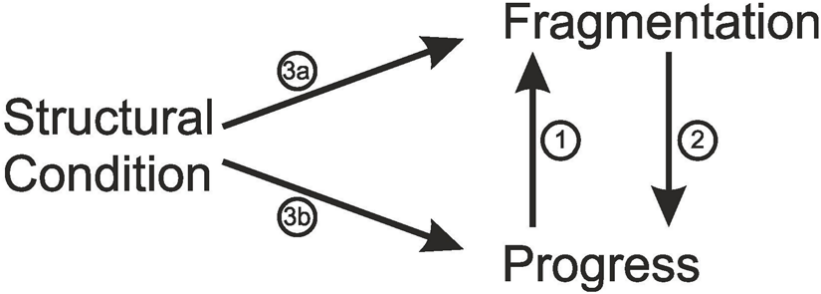
Possible explanations for the correlation of scientific progress and fragmentation. Each arrow represents one of the causal relationships that we study.

Here, we study the question why disciplinary fragmentation and limited scientific progress appear to coincide often. Fig. 1 illustrates three possible causal processes that are able to explain this observation. First, progress might causally affect fragmentation, which is illustrated by Arrow 1 in the figure. For instance, it is is possible that the natural sciences are less fragmented into opposing schools because they have developed a scientific consensus very early in their history [5]. Second, as suggested by Arrow 2, a high degree of fragmentation might slow down scientific progress, for instance because fragmentation hampers the diffusion of ideas and insights [5]. This is in line with the position held by philosopher of science T. Kuhn, according to whom the simultaneous co-existence of opposite schools of thought is symptomatic for a pre-paradigmatic science. In this state, the lack of shared fundamentals is a concrete obstacle to collective progress [14]. Third, fragmentation and progress might not influence each other, but there might be other variables that affect both outcomes (see Arrows 3a and 3b). For instance, scarcity of public funding might not only slow down progress but also further increase competition between scientists and hamper their willingness to interact with competing research teams, which in turn fosters the formation of distinct clusters [15].

Here, we present a general theory about the behavior of members of a scientific discipline, and use this theory to develop testable hypotheses about which of the causal processes shown in Fig. 1 is actually responsible for the observation that fragmentation and progress are correlated. It has been demonstrated that formal modeling and computational methods are invaluable tools to analyze such complex theories and identify predictions that might have been overlooked without a formal analysis [16]. Accordingly, we developed an agent-based model of scientific discovery, which was inspired by earlier formal models of the scientific system [17–23]. The model makes very few assumptions about the behavior of the individual scientists, and it assumes that scientists in all fields react in the same way to the circumstances in their respective field. The model reflects the conceptions of science that the two quotes illustrate, scientific realism and constructionism. On the one hand, scientists make positive observations of reality, which influences their scientific approach and explanation of the real world. On the other hand, scientists influence each other’s approaches and theories, which is the central mechanisms of social construction.

## The Formal Model

The theory is built around a research problem that a population of *N* = 100 scientists (agents) seeks to solve. This research problem is described by two real-valued variables, which create a two-dimensional epistemic space of possible answers, which scientists explore. We chose to model only two dimensions because it is computationally efficient, and easy to visualize [24]. However, the model could also be extended to study more complex spaces. The “true” answer to the research problem is located in the center of this two-dimensional space. We call this location the “*ground truth*”.

At any moment in time *t*, each scientist *i* holds a certain *view*, i.e. his or her current answer to the research problem. This view is defined by the two coordinates that describe his or her position *x*_*i*_ in the two-dimensional epistemic space. In addition, each scientist is moving through the epistemic space, exploring possible answers to the research question. We call the vector ${\vec{v}}_{i}\;$ describing this movement the *approach* of the scientist. In sum, each scientist is described by three variables: (1) his or her current position, (2) the direction of exploration, and (3) the speed at which they move in the space of possible answers. That is, some scientists explore the space in rather small steps, because they feel that the true value is close to their current view. Others may explore at a much higher pace.

At the outset of the dynamics at time *t*_0_, we implemented that all agents adopt a random position within a square of size 1 with the ground truth located in the center of the square. This square, however, does not limit the space of possible answers to the research problem, as agents can adjust their views in any direction, exploring answers that are outside of the range of initial views. Likewise, we assumed that agents initially adopt a random approach ${\vec{v}}_{i}\left(t_{0}\right)$ drawn from a uniform distribution. The size of the vector ${\vec{v}}_{i}\left(t_{0}\right)$ is bounded between −0.5 and +0.5. That is, at the outset each agent explores the epistemic space in a random direction and with a random speed.

At each point in time *t*, the computer program executes a random sequential update of the positions of all agents. To be more precise, the updated position is a function of the previous position and the approach. However, before the new position is determined, the computer program updates the approach of agent *i* based on Equation (1), which contains three terms representing the three forces that influence the position of the scientist.

$$\frac{d{\vec{x}}_{i}}{dt}=\underset{Ground\;truth}{\underset{︸}{{\vec{C}}_{h}\left(t\right)}}\;+\;\underset{Social\;influence}{\underset{︸}{{\vec{v}}_{i}\left(t\right)}}\;+\;\underset{Noise}{\underset{︸}{{\vec{\xi }}_{i}\left(t\right)}}$$ (1)

The first term of Equation (1) reflects the attraction ${\vec{C}}_{h}\left(t\right)$ by the ground truth, the fixed point mentioned by Weinberg in his quote [1]. As discussed in existing literature of epistemic simulations with a ground-truth [22], this term represents the feedback that individuals receive from the results of their research. Formally, ${\vec{C}}_{h}\left(t\right)$ is modeled as:

$${\vec{C}}_{h}\;\left(t\right)\;=\;\frac{{\vec{x}}_{h}-{\vec{x}}_{i}\;\left(t\right)}{\tau }$$ (2)

where ${\vec{x}}_{h}\;-\;{\vec{x}}_{i}\left(t\right)$ is the distance between agent *i*’s view and the location of the ground truth. This implies that agents with views that are very distant from the truth receive a stronger pull back towards the ground truth. The parameter *τ* allows to control the strength of the pull.

The second term in Equation (1) includes social influence, the social process of construction that Feyerabend referred to in his quote [2]. It assumes that agents tend to adopt approaches that are equal to the average of those scientists whose views are sufficiently similar [25, 26]. The degree to which social influence affects the approach of the agents is controlled by two parameters, as shown in Equation (3). First, parameter *R*, called the “interaction radius”, determines the maximal distance in space for two agents to influence each other. In other words, if the distance between the views of two agents exceeds the value of *R*, then there is no social influence. Second, parameter *α* allows to manipulate the strength of social influence. Larger values of *α*, with 0 < *α* < 1, implement that agents are more open to influence.

$${\vec{v}}_{i}\;\left(t\;+\;1\right)\;=\;\left(1-\alpha \right)\;{\vec{v}}_{i}\;\left(t\right)\;+\;\alpha \;{\langle {\vec{v}}_{j}\;\left(t\right)\rangle }_{R}$$ (3)

The third ingredient of Equation (1) is the noise term ${\vec{\xi }}_{i}\left(t\right)$, which models that agent *i*’s approach does not deterministically depend on social influence and the ground truth. In contrast, measurement errors or random external influences can lead agents to sometimes deviate from the approach that these deterministic forces imply. Technically, this so called “angular noise” is modeled as a random perturbation to the direction of the approach, while the speed of the approach stays unchanged. The random value is drawn from a normal distribution with an average of zero and a standard deviation of *σ*. The latter is a further parameter and allows to control to which degree the approach of the agents is affected by random deviations. In addition, we implemented so called “position noise”, which does not affect the approach of the agent, but is added to her current view after it has been updated. Also the position noise is randomly drawn from a normal distribution, with an average of zero and a standard deviation of *ε*.

### Model Predictions

It is difficult to derive precise predictions about the effects of the model parameters on the progress of scientific disciplines and their tendency to fragment, when the three forces operate simultaneously.

Intuitively, one would expect that the attraction force by ground-truth should eventually allow all scientists to solve the scientific problem and adopt a view that coincides with the truth. However, scientists might also adopt approaches that lead them away from the truth. In this case, the force exerted by the truth must be very strong to convince them to adjust their direction of exploration, or those scientists may never reach the truth, being mislead by their own approach.

Moreover, it is not clear whether social influence speeds up or slows down the process of identifying the truth. On the one hand, one might expect that in a discipline where all scientists are affected by the truth, social influence will intensify the impact of the truth on the view of each individual scientist, increasing the speed of the truth finding process. In fact, classical models of influence processes in networks have demonstrated that when no subset of individuals is entirely disconnected from the others, social influence will eventually lead to the formation of a global consensus [27–32]. In addition, if at least some scientists are influenced by the truth, one would expect that the consensus will converge on the truth. On the other hand, social influence might have the opposite effect, obscuring the signal of the truth, and making it more difficult to identify the correct answer to the research problem. What is more, empirical research has identified homophily as a very strong force in human populations [33]. Homophily, the tendency of birds of a feather to flock together, has been implemented in social influence models with the assumption that individuals are only influenced by others who are sufficiently similar [22, 26]. If social influence acts in tandem with homophily, populations can fall apart into distinct subgroups. Such fragmentation dynamics might further slow down scientific progress.

Finally, also the effects of noise are difficult to understand. On the one hand, noise disturbs the influence of the truth and should thus slow down scientific progress. On the other hand, noise has been shown to decrease the tendency of populations to fragment [34], which might speed up progress. Then again, noise can also guide scientists who got stuck with their approach back on the right track.

To explore in detail the effect of the three forces acting simultaneously, we performed agent-based computer simulations, and we describe the resulting model dynamics in the next section.

### Model Dynamics

**Fig 2:**
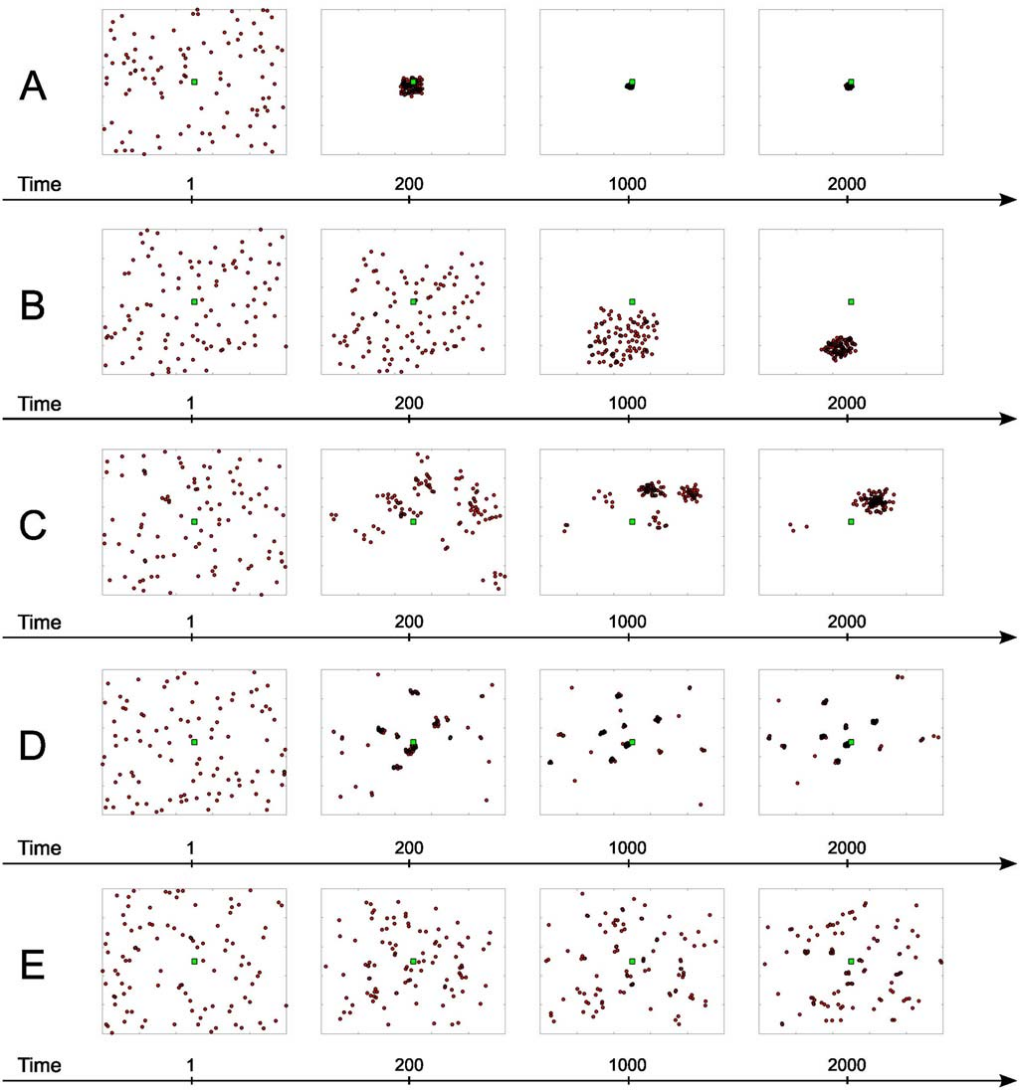
Ideal typical runs showing different patterns of consensus and level of scientific progress at four time steps: *t* = 1, 200, 1000, 2000. Panel **A** shows a steady trend towards consensus ending up almost perfectly on the ground truth [*R* = 0.3, *α* = 0.5, *τ* = 0.9, *σ* = 0.01, *ε* = 0.1]. In Panel **B**, the same process of convergence ends up far away from truth [*R* = 0.3, *α* = 0.5, *τ* = 10, *σ* = 0.01, *ε* = 0.1]. Panel **C** shows the emergence of different schools of thought, that gradually merge together in proximity of the truth [*R* = 0.06, *α* = 0.9, *τ* = 3, *σ* = 0.02, *ε* = 0.25]. Panel **D** shows the emergence of many small clusters that persist over time[*R* = 0.03, *α* = 0.9, *τ* = 1, *σ* = 0.01, *ε* = 0.1]. Finally, in Panel **E** the process of school formation is so slow that even at the end of the simulation a relevant share of agents is still isolated [*R* = 0.03, *α* = 0.01, *τ* = 1, *σ* = 0.01, *ε* = 0.1].

The interplay of the decisions that the simulated scientists make can generate very different patterns of disciplinary fragmentation and scientific progress. For illustration, Fig. 2 shows five very different yet typical simulation runs. In each run one can observe a distinct collective dynamic, even though all five runs started with a very similar random initial distributions of individual views and approaches. The run shown in Panel A was characterized by a very strong tendency to converge onto the ground truth at the center of the epistemic space. In this run, fragmentation was very low and progress was quick. In the run shown in Panel B, agents’ views also converged, but the emergent consensus was substantially further apart from the ground truth. Thus, progress was more limited in this run. Panel C shows a run where we observed fragmentation. That is, several internally homogeneous schools of thought with mutually different views emerged. As the simulation progressed, however, the schools merged and collectively progressed towards the ground truth. Similar dynamics were observed in the run shown in Panel D. However, the state of fragmentation was much more robust and progress slower. Finally, Panel E illustrates a run where the fragmentation was even stronger and almost no progress occurred. Additional patterns of collective exploration are shown in S1 Model Dynamics Video.

**Fig 3:**
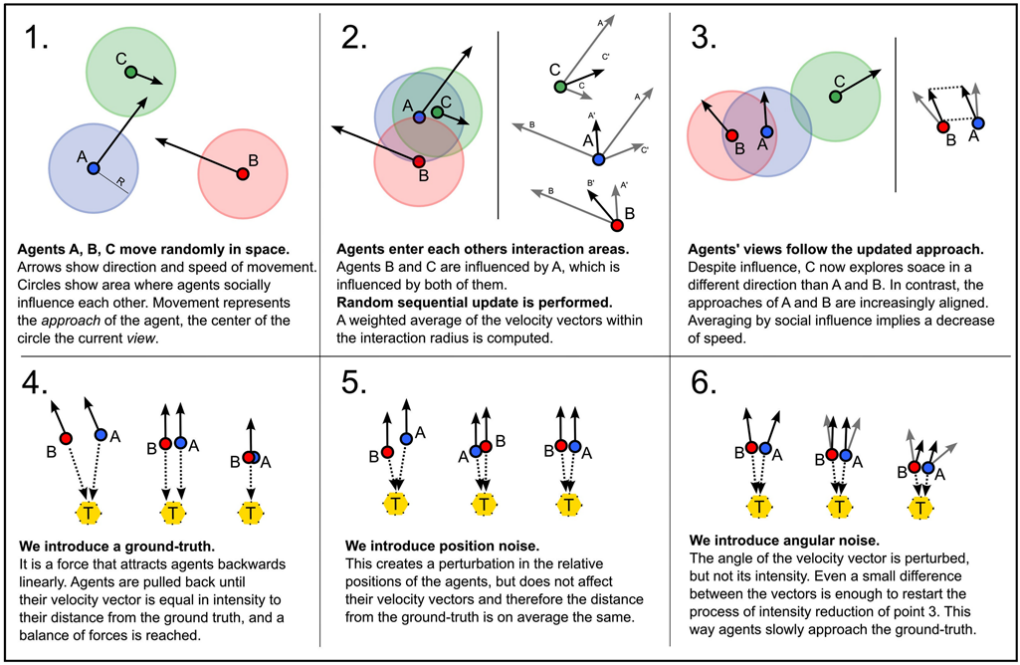
Summary of model dynamics. How the movement of agents in the epistemic space is affected by social influence (panels 2 and 3), attraction to ground truth (panel 4), and noise (panels 5 and 6).

The collective dynamics that the model generates are graphically summarized in Fig. 3, and can more formally be described as follows. Let us start with the case where agents’ approaches are only influenced by the ground truth (*α* = *σ* = *ε* = 0). In this case, each individual agent is exploring the epistemic space independently from the others. This movement, however, slows down and eventually stops when an agent reaches a point in the epistemic space where the strength of attraction towards the ground truth perfectly equals the absolute speed of exploration. In this situation, the force towards the truth points into the opposite direction of the agent’s approach. This balance between one’s own approach, and the information received from measurements completely determines the final position of the agent. On the collective level, the system reaches a state of equilibrium when all agents have stopped exploring the epistemic space. No consensus can be built under these conditions, and overall progress is limited.

However, social influence (*α* > 0) can improve the situation. In the presence of social influence, agents adopt approaches and views that are similar to others, which leads to the formation of clusters. Within each cluster, agents have similar views but their approaches will initially point into different and sometimes even opposite directions. As a result of social influence, these differences in agents’ approaches are averaged out until all cluster members have adopted exactly the same approach. Averaging by social influence implies that the absolute exploration speed of the agents in the cluster is decreased. As a striking consequence, the point where the force of their approach is equal to the attraction by the ground truth is closer to the truth. For this reason, social influence facilitates progress. Nevertheless, after an initial loss in absolute speed, agents manage to perfectly align their approaches within a cluster. From that moment on, their speed will remain constant. In fact, the system is very likely to reach a state of equilibrium where the population consists of multiple homogeneous but mutually distant clusters with views that can be very far away from the ground truth.

Furthermore, angular noise can facilitate progress. As a result of angular noise, members of a cluster sometimes adopt approaches that deviate from the approaches of other cluster members. These deviations are random and, thus, lead to adjustments of agents’ approaches in random directions. In the long run, these deviations are averaged out by the social influence process. However, the deviations also modify the initial approach of the cluster and eventually lead to a further deceleration of the cluster. This, in turn, allows clusters to move closer to the ground truth.

Position noise has different effects than the angular noise, as it does not affect agents’ approaches but their views. As a consequence, even when agents have adopted identical approaches, position noise results in variation of views within a cluster of agents.

## Simulation Experiments

The overarching research question of this study is why disciplinary fragmentation and scientific progress are correlated. We, therefore, conducted two simulation experiments exploring which of the causal processes illustrated in Fig. 1 are responsible for this correlation according to our formal model.

Experiment 1 focuses on the effects of structural conditions on fragmentation (Arrow 3a) and progress (Arrow 3b). To this end, we manipulated the model parameters that determine the strength of the three forces guiding scientists behavior in our model and measured how this affected model predictions. In other words, we studied whether the attraction to the truth, social influence, and noise affect progress and fragmentation.

Experiment 2 studied to which degree progress affects fragmentation (Arrow 1) and vice versa (Arrow 2). To this end, we experimentally manipulated the degree to which the simulated populations of researchers are fragmented and how distant their views are from the truth already at the beginning of the simulated research process. Our question was, whether the initial degrees of fragmentation and progress affected subsequent degrees of progress and fragmentation.

In all simulation experiments we studied two outcome measures to quantify the structural patterns of disciplinary fragmentation and progress that the model generates. To measure the discipline’s progress at a given point in time, we calculated the average absolute Euclidean distance between agents’ current views and the ground truth. Obviously, higher values indicate a limited degree of scientific progress. As a measure of the degree of fragmentation, we focused on the number of clusters in the population. Further details about the implementation of the simulation experiments are available in S2 Supporting Information.

### Simulation Experiment 1: Effects of Parameters on Fragmentation and Progress

Experiment 1 explored the effects of social influence, attraction by the ground truth, and noise on the degree of fragmentation and progress in the scientific discipline—Arrows 3a and 3b in Fig. 1.

#### Effects of social influence

**Fig 4:**
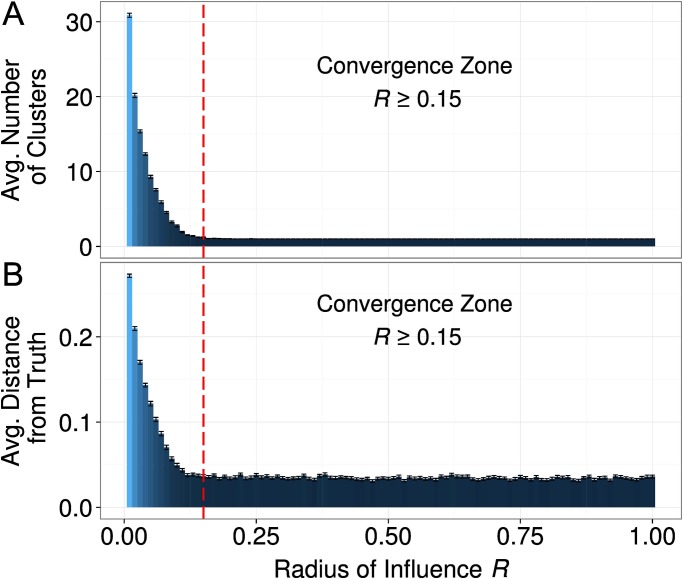
(A) The effect of the influence radius *R* on the average number of clusters, and (B) the average distance from the ground truth at time point 2,000. We identified a convergence zone (*R* > = 0.15) where dynamics always generated consensus in close proximity of the ground-truth. Error bars represent standard errors of the mean. [*R* = (0.01 − 1), *α* = 0.5, *τ* = 1, *σ* = 0.01, *ε* = 0.1]

The bar graphs shown in Fig. 4 depict that the interaction radius *R* had profound effects on fragmentation and progress. It turned out that after 2,000 time steps agents almost always developed a global consensus whenever the interaction radius was equal or greater than 0.15 (Panel A). However, a more limited interaction radius fostered the formation of fragmented clusters. What is more, Fig. 4 Panel B shows that the simulated disciplines with a large influence radius were characterized by a consensus on a view that was close to the ground truth. The average distance from the truth was substantial, however, whenever agents had a small influence radius and had formed clusters.

Having found the very strong effect of the interaction radius *R*, we decided to always report two cases for the analyses of the remaining parameters: (i) a small influence radius of *R* = 0.03; and (ii) a large influence radius of *R* = 0.3.

**Fig 5:**
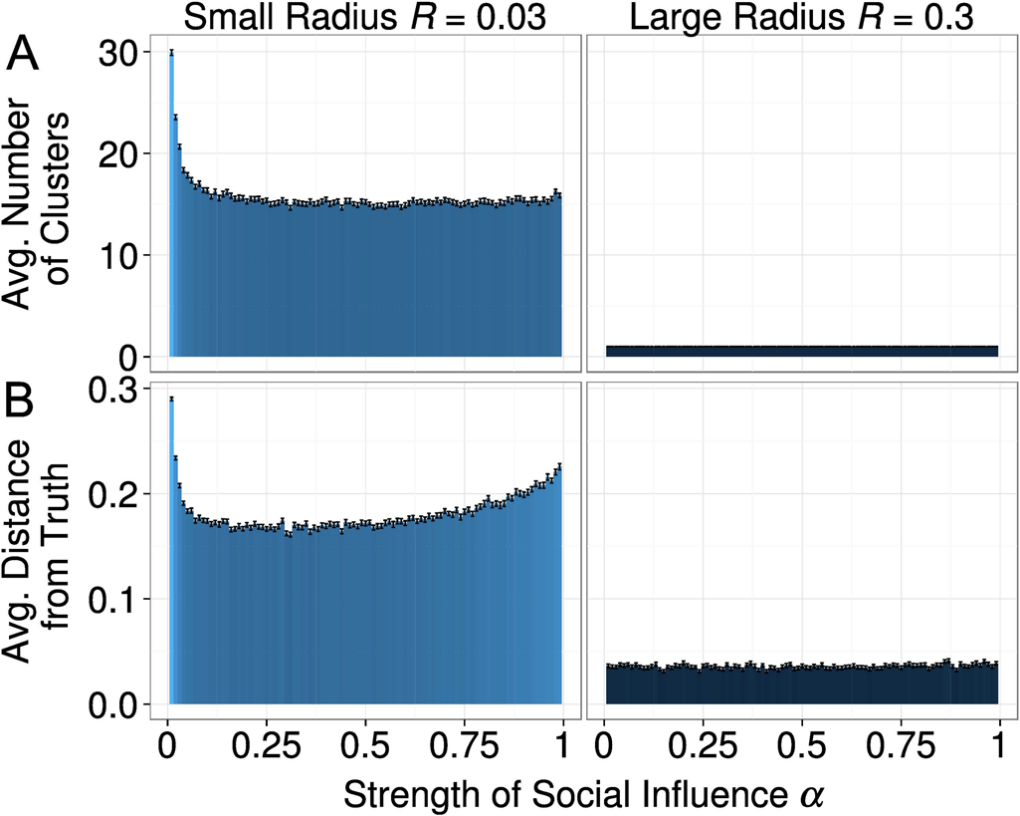
(A) The effect of the strength of social influence *α* on the average number of clusters, and (B) the average distance from the ground truth at time point 2,000. The effect of *α* is particularly strong for *R* = 0.03, and negligible for *R* = 0.3. In the case of a small radius of interaction, the U-shaped relationship depicted in Panel B is due to the high level of cohesiveness of the clusters at the beginning of the simulation, when social influence is very high. Error bars represent standard errors of the mean. [*R* = (0.03; 0.3), *α* = (0.01 − 0.99), *τ* = 1, *σ* = 0.01, *ε* = 0.1]

Fig. 5 informs about the effects of the second social-influence parameter of our model, the strength *α* of social influence. Panel A shows that the strength of social influence had an effect when the influence radius was small (*R* = 0.03). Under this condition, very weak social influence resulted in a higher degree of fragmentation. For instance, when *α* adopted a value of 0.01, the smallest value that we studied, we counted 30.2 clusters on average. When *α* was increased to 0.1, we counted only 15.9 clusters on average. However, when social influence was stronger than *α* = 0.25, a further increase in social influence strength hardly affected fragmentation. The populations with a large influence radius (*R* = 0.3) always reached a consensus within the 2.000 simulation events. However, when fragmentation was measured earlier in the simulation process (e.g. *t* = 100, not shown here) a similar effect obtains as for the simulations with the small influence radius.

**Fig 6:**
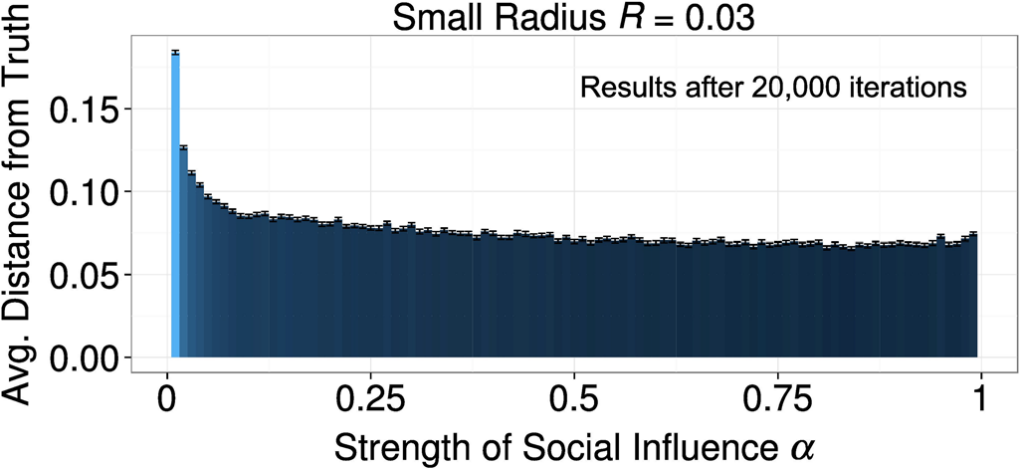
The effect of the strength of social influence *α* on the average distance from the ground truth at time point 20,000. The U-shaped relationship depicted in Fig. 5 disappeared, and only low values of alpha cause a slower scientific progress. Error bars represent standard errors of the mean. [*R* = (0.03; 0.3), *α* = (0.01 − 0.99), *τ* = 1, *σ* = 0.01, *ε* = 0.1]

Panel B suggests that the strength of social influence had a non-linear, U-shaped, relationship with scientific progress. As explained in Section Model Dynamics, social influence leads to a reduction in the absolute exploration speed through a process of reciprocal adjustment of the approaches of the agents in the same cluster. Therefore, when social influence is very weak, it is not surprising that scientific progress is slower. However, that also very high values of the parameter *α* can retard scientific progress was completely unexpected. This apparent anomaly can be explained as follows. A very strong social influence create very cohesive clusters very early in the simulation. We measured the average pairwise distance between agents in the biggest cluster, and it turned out to be significantly lower for very high values of *α*. The difference is small, but it counts for about one third of the size of the small interaction radius (*R* = 0.03). It is therefore enough to significantly reduce the process of merging of clusters at the beginning of the simulation. This leads to a lower average cluster size at the beginning of the simulated discipline, which in turn is the cause of the accumulated scientific retard measured after 2,000 iterations. On the other hand, such a gap is actually not permanent. If we let the simulations run for 20,000 iterations, we observe an approximately homogeneous degree of scientific progress for any value of *α* > 0.1 (see Fig. 6). Therefore, only a very weak social influence can slow down progress in a scientific field for an extended period of time.

In sum, social influence turned out to foster consensus formation and progress. In the simulations with a very weak social influence we found substantially more fragmentation than in runs with stronger social influence. What is more, the model predicts a very strong effect of the influence radius *R*. When agents are influenced only by very similar peers, we observed much more fragmentation and limited progress than in populations where also others with dissimilar views have an influence on the view and approach of the individual scientist.

#### Effects of Noise

In Section Model Dynamics we argued that angular noise, in tandem with social influence, slows down the movement of clusters, which in turn increases the relative impact of the ground truth on agents’ views. As a consequence, angular noise fosters progress. In addition, the random movement of clusters that is caused by the angular noise facilitates the merging of clusters that would have remained distant without randomness. Thus, angular noise also decreases disciplinary fragmentation.

**Fig 7:**
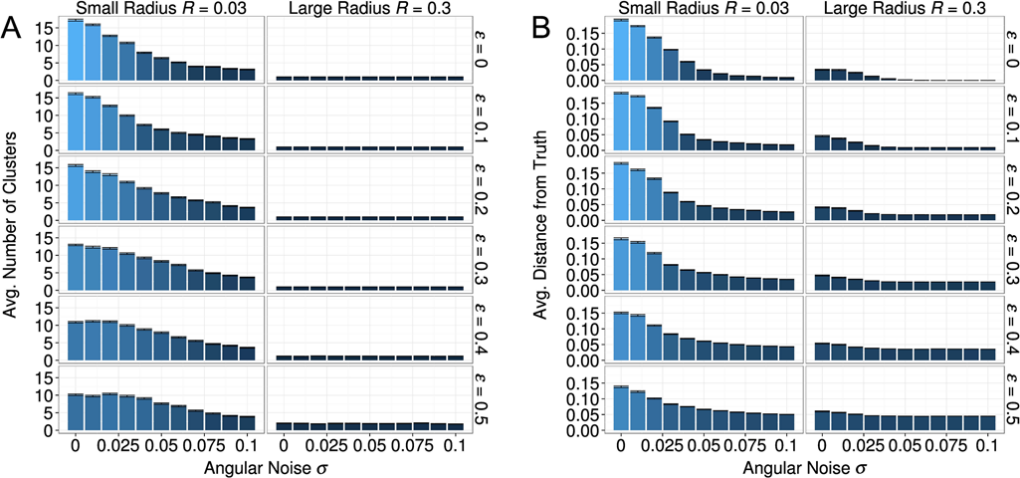
Average number of clusters (A), and average distance from ground-truth (B) for each combination of position noise (rows) and angular noise (x-axis within each row). Angular noise is responsible for most of the variation of on both outcome measures, however higher values of position noise attenuate its effect. Error bars represent standard errors of the mean. [*R* = (0.03; 0.3), *α* = 0.5, *τ* = 1, *σ* = (0 − 0.05), *ε* = (0 − 0.5)]

Fig. 7 illustrates how position noise *ε* and angular noise *σ* affected fragmentation and progress. The figure shows that increased levels of either form of noise resulted in fewer clusters and views that were closer to the truth. These effects were most pronounced in the simulations with small influence radiuses, because simulated disciplines with large radiuses basically always ended with a global consensus close to the ground truth.

The effect of position noise on fragmentation and progress turned out to be relatively weak, compared to the strong effects of angular noise. One reason is that position noise only creates new diversity in views. When position noise is very strong, this can motivate some agents to adopt views that are relatively far away form the average of the population and can even lead to the formation of small clusters.

#### Effects of the Attraction to Ground Truth

**Fig 8:**
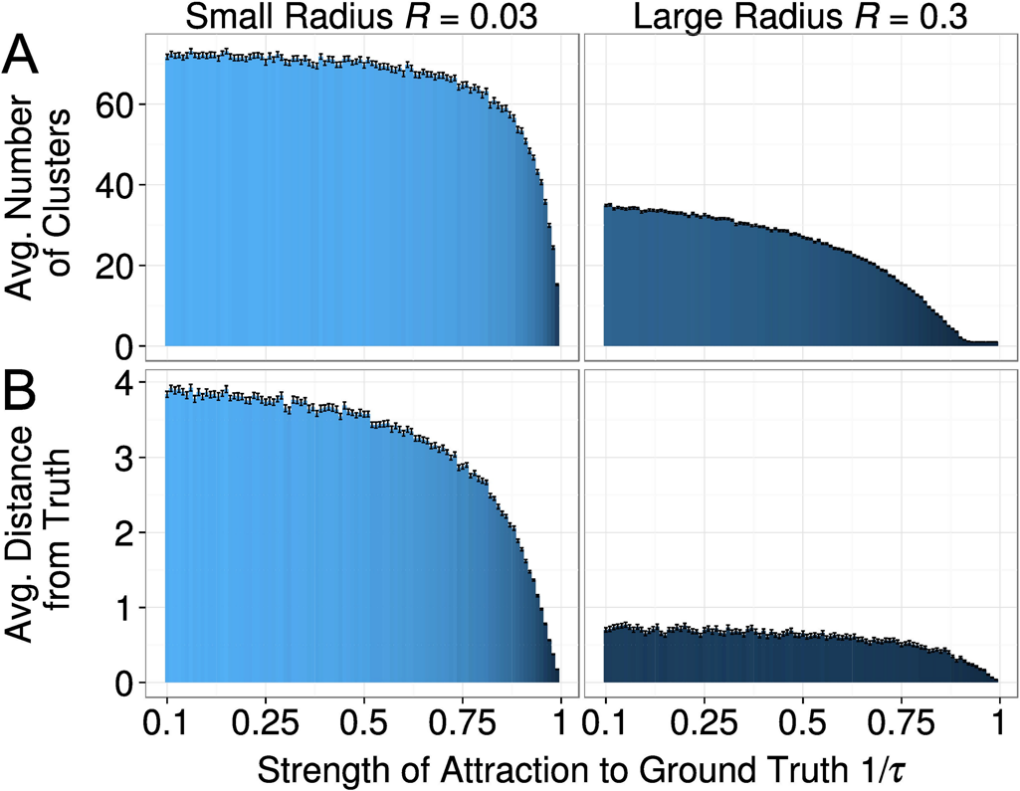
The effect of the strength *τ* of attraction to ground truth on the average number of clusters (A), and the average distance from the ground truth (B) at time point 2,000. In general, the weaker the attraction (higher values of *τ*) the more clusters the further away. However, most of the variation happens for values of *τ* between 1 and 20. Error bars represent standard errors of the mean. [*R* = (0.03; 0.3), *α* = 0.5, *τ* = (1 − 100), *σ* = 0.01, *ε* = 0.1]

Fig. 8 shows how fragmentation and progress are affected by changes in the strength *τ* of attraction to the ground truth. As expected, we found that fragmentation was weaker and progress faster when the truth had a stronger impact on agents’ research approaches. In fact, the stronger the strength of attraction, the closer the clusters move towards the ground-truth. Being pulled in the same direction, clusters may develop views that are similar enough to lead to social influence between members of different clusters and, eventually, a merging into one big cluster. Within the joint cluster, agents develop a new shared approach and their speed decreases, which again lets them move closer to the ground-truth and increases the chances of merging further. As the merging of clusters is less likely when the influence radius is small, this self-reinforcing dynamics does not obtain when agents have small influence radii. This is why, populations with small influence radii always experience more fragmentation and more limited progress for any value of *τ*. This result is consistent with what we have shown in the previous sections, and holds true even when the ground-truth is maximally weak, i.e. *τ* = 100.

Furthermore, Fig. 8 also shows that most of the variation in both outcome variables take place already for a small range of values of *τ*, meaning that a small variation in the strength of attraction to the ground-truth can lead to big macro-level changes in the outcome variables.

In conclusion, both progress and fragmentation were substantially affected by parameter *τ*. The stronger agents were affected by the ground truth, the closer their views were to the ground truth, and the less fragmented the field was.

#### Correlation between Fragmentation and Progress in Experiment 1

**Fig 9:**
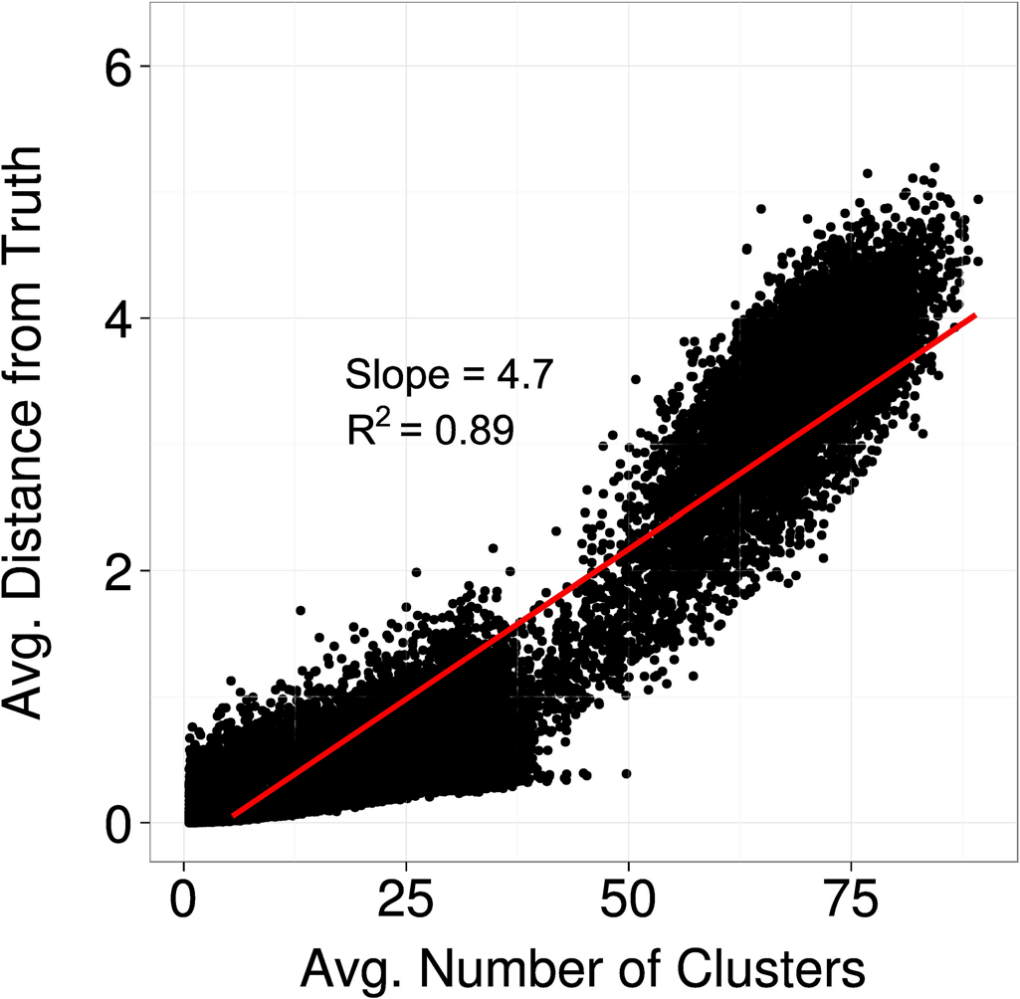
Scatter plot clustering vs progress. All data points are observations produced by Experiment 1.

Fig. 9 informs about the degree to which fragmentation and progress correlated in Experiment 1. Each dot of the scatter plot shows the degree of fragmentation and progress at the end of one simulation run. One can see that those runs where the disciplines consisted of many fragmented clusters tended to be characterized by limited progress (high average distance from truth). This shows that the formal model is able to replicate the empirical observation that progress and fragmentation are correlated.

### Simulation Experiment 2: Relationships between Fragmentation and Progress

Experiment 2 tested whether or not fragmentation and progress influence each other—Arrows 3a and 3b in Fig. 1. To this end, we experimentally manipulated the initial degrees of fragmentation and progress in the simulated disciplines and tested whether this affected subsequent levels of the two outcome variables.

#### Effects of Initial Fragmentation on Progress

**Fig 10:**
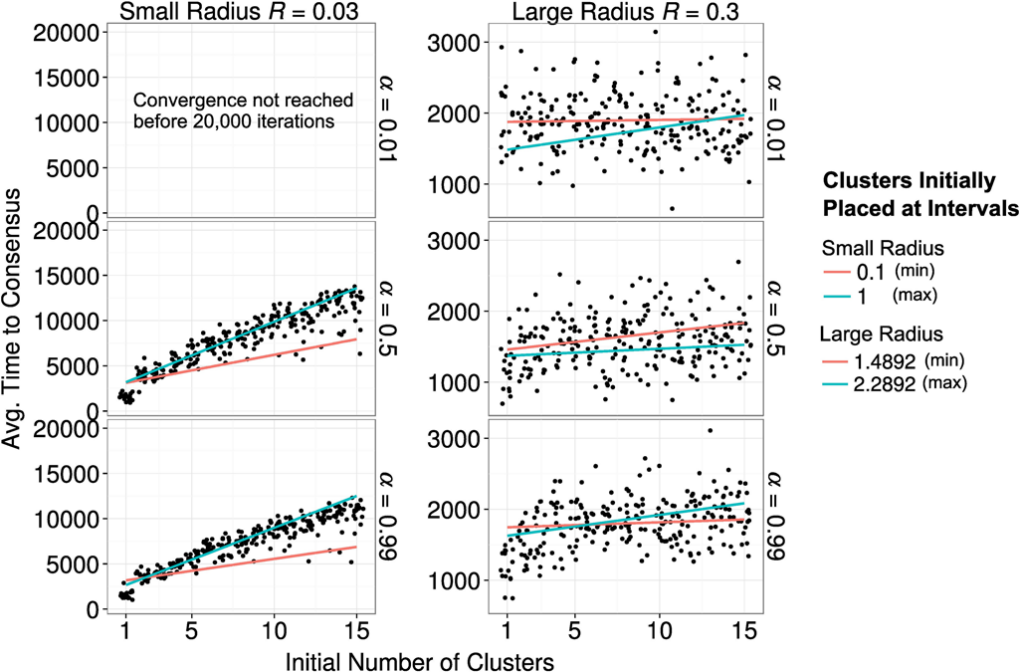
The effects of fragmentation on progress. At the beginning of the simulation agents were randomly preassigned to *c* = (1..15) clusters. Each group of agent was then placed at a fixed distance from the ground truth, and equidistant from the neighboring groups. We varied the distance between 0.2 and 1 in steps of 0.05 units for a small radius of interaction, and between 1.4892 and 2.2892 for a large radius of interaction. Graph shows the time necessary to 75% of the agents to end up in a radius of 0.05 units from truth. Simulations were stopped after 20,000 iterations. In general, a clustered field takes longer to build consensus, however the relationship is much more marked when interaction radius is small. On top of this, if also social influence is also weak, e.g. *α* = 0.01, reaching a consensus in proximity of the truth becomes virtually impossible. [*R* = (0.03, 0.3), *α* = (0.01, 0.5, 0.99), *τ* = 1, *σ* = 0.01, *ε* = 0.1]

We measured scientific progress in the simulated disciplines in terms of the time necessary to achieve a stable state of *consensus* on the ground truth, where 75 percent of the agents had adopted a view that deviated not more than 0.05 units from the ground truth. We terminated all simulations after twenty thousand iterations. In case some runs did not reach a state where 75 percent of the agents agreed on a view close to the ground truth within this time frame, we set our outcome measure to the value of 20,000. This happened mainly under one experimental condition, namely when the influence radius *R* was small and the strength of social influence *α* was very weak (see Fig. 10). As a consequence, we are confident that the decision to terminate these simulations did not alter our results.

As the initial degree of fragmentation was the independent variable of these analyses, we included only runs with fewer than 16 initial clusters, in order to guarantee that clusters were perfectly isolated from each other at the outset of the simulation runs.

Fig. 10 summarizes the core results, showing a jittered scatter plot where each dot represents the average number of iterations needed to reach a 75 percent consensus on the ground truth. The subpanels show the results for different influence radii *R* and different strengths *α* of social influence. Note that we used a different scale for the y-axes on the left panels than for the right panels. In addition, the blue (red) lines show regression estimates for those runs with the maximal (minimal) initial distance from the ground truth.

Fig. 10 shows that there were two different consensus regimes, depending on the interaction radius *R*. In the case of a small radius (*R* = 0.03), there is a significant positive relationship between the number of initial clusters and the time necessary for reaching consensus. In the case of large radius (*R* = 0.3), we found a similar relationship, but much weaker. This difference results from the very strong tendency towards consensus that a large influence radius *R* implies. This tendency is so strong that the initial degree of fragmentation from the truth can only have a limited effect on the amount of time needed to reach a consensus on the true value.

It turned out that the simulated disciplines with a small influence radius (*R* = 0.03) and weak social influence (*α* = 0.01) never reached a state were at least 75 percent of the agents adopted views close to the ground truth, independently of the initial degree of fragmentation. Thus, even when there is a consensus already at the outset (1 cluster), progress was very limited when social influence was very weak.

**Fig 11:**
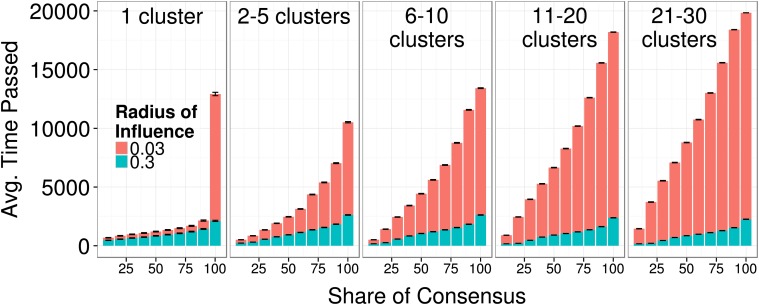
Distributions of consensus shares *X*(10, .., 100) under different initial clustering conditions. A field with a large radius of interaction can sustain a faster consensus growth for *any* intermediate share of consensus. Interestingly, if the interaction radius is small and agents are initially placed in a single cluster, it is extremely hard to reach full consensus (100% consensus share) due the to reduced social influence effects on those agents that manage to leave the initial cluster. Error bars represent standard errors of the mean. [*R* = (0.03, 0.3), *α* = (0.5, 0.99), *τ* = 1, *σ* = 0.01, *ε* = 0.1]

Fig. 11 provides more detailed information, plotting the distribution of consensus shares over time, i.e. the time necessary to build a consensus of *X*% of agents within a radius of 0.05 units from the ground truth. The figure confirms the earlier findings that disciplines with a large influence radius *R* reach consensus faster than populations of agents with small radii. Keeping the number of initial clusters constant, a field with a larger radius of interaction can sustain a faster consensus growth for *any* intermediate share of consensus. In addition, even if all agents are initially placed in one single cluster away from the truth, those that are equipped with a smaller radius of interaction (see red bars) require more time to collectively find the ground-truth. Moreover, if the interaction radius is small and agents are initially placed in a single cluster, it is extremely hard to reach a perfect consensus (100% consensus share). It takes even longer than if agents are initially split up into more clusters (2 to 5). This counter-intuitive finding is obtained because noise can lead some agents to leave their initial group. With a small influence radius, these isolated agents will remain isolated until they happen to join another cluster. This, however, can last very long or may never happen when there are very few clusters initially.

#### Effects of Initial Progress on Fragmentation

In order to measure the degree of fragmentation, we counted the number of clusters at the point in time when 50 percent of the agents had adopted a view not further than 0.05 units away from the ground truth. We refrained from measuring fragmentation at some predefined number of iterations, because in some runs perfect consensus was reached very quickly, while in others lasted very long. We were, however, interested in the degree of fragmentation during the process of reaching the consensus. Furthermore, it turned out that 50 percent was a good threshold, as studying smaller shares implied that the initial degree of fragmentation basically explained the biggest part of the variance in later degrees of fragmentation. Larger values than 50 percent, on the other hand, imply that the theoretically possible number of clusters was reduced, which led to undesirably small variations in the outcome variable.

**Fig 12:**
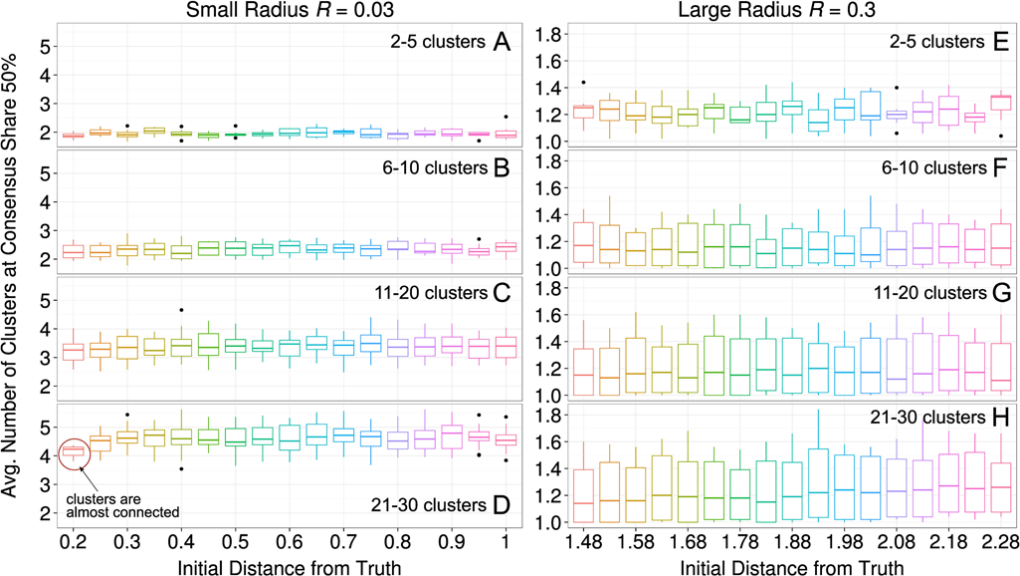
The effects of progress on fragmentation. At the beginning of the simulation agents were randomly preassigned to *c* = (1..30) clusters. Each group of agent was then placed at a fixed distance from the ground truth, and equidistant from the neighboring groups. Panels A-D show results for a small radius *R* of interaction *R* = 0.03, and panels E-H for a large radius of interaction *R* = 0.3. We varied the initial distance from ground truth between 0.2 and 1 in steps of 0.05 units for a small radius of interaction, and between 1.4892 and 2.2892 for a large radius of interaction. We have then measured the number of clusters when a consensus share of 50% was reached. Results show that the average number of clusters is largely independent from the initial level of progress. In the case of a small radius, it is heavily determined by the initial number of clusters. Only in panel D (21 to 30 clusters), the first boxplot is lower than the rest because clusters are almost all connected at a distance of 0.2. [*R* = 0.03, *α* = (0.5, 0.99), *τ* = 1, *σ* = 0.01, *ε* = 0.1]

Fig. 12 shows the result of this experiment. All subpanels depict the same pattern: the degree of fragmentation was hardly affected by the initial distance from the ground truth. In other words, we found that initial progress did not affect the degree of fragmentation in later stages of the simulations.

Panel D in Fig. 12 shows that populations that consisted of many clusters that were initially already close to the truth experienced smaller amounts of fragmentation than equally fragmented populations with initial views that deviated more from the truth. This difference is significant. However, this result is driven by the fact that these populations were not sufficiently fragmented at the outset of the simulation, i.e. were already clusters forming one connected component.

## Mediation Analysis

Experiment 1 showed that the social influence radius *R*, the strength of angular noise *σ*, and the strength of the attraction to the ground truth *τ* had significant effects on both fragmentation and progress. Experiment 2 showed, in addition, that fragmentation decreases progress, while progress has no effect on fragmentation. For the purpose of our study, it is crucial to check whether the effect of the three independent variables on progress is mediated by fragmentation. In other words, it is possible that the three independent variables studied in Experiment 1 are acting directly only on fragmentation, and by reducing it, they have an indirect effect on progress. We, therefore, implemented Baron and Kenny’s mediation test [35] on the data of Experiment 1 for each of the parameters *R*, *σ*, and *τ*. Further details about the mediation analysis are available in S2 Supporting Information.

The mediation analysis consisted of three steps. First, we estimated a linear regression with progress as the dependent variable and one parameter as the independent variable. Experiment 1 already demonstrated that the three parameters (*R*, *σ*, and *τ*) correlated significantly with progress. Second, we regressed fragmentation on the respective parameter. Once more, Experiment 1 has shown that the effect was present. Third, progress was regressed on fragmentation and the respective parameter. If the size of the statistical effect in Step 3 was smaller than in Step 1, we concluded that the parameter has both a direct and an indirect effect on progress. If the statistical effect even turned insignificant in the regression of Step 3, then we concluded that the effect of the parameter on progress is completely mediated by fragmentation.

Table 1 shows the results of the mediation analyses. Comparing the regression coefficients of the respective model parameter in the regressions of Step 1 and Step 3 (see numbers printed in bold), one can see that the effects of all three variables on progress are at least partly mediated by fragmentation. Moreover, the effect of angular noise on progress even turned out to be completely mediated.

The influence radius *R* had a very strong effect on progress (see Fig. 4). The mediation analysis showed, however, that 92.4 percent of the total effect of *R* on progress is mediated by fragmentation. According to the Preacher and Hayes test, this mediation is significant (coefficient = -.05, with a standard error of .002). As Fig. 4 showed highly nonlinear effects of the influence radius on progress and fragmentation, we replicated the mediation analysis with only those simulation runs that had radii below or equal *R* = 0.1. In turned out that in this subset of the simulations 69.5 percent of the effect of the influence radius *R* on progress was mediated by fragmentation. Thus, also the direct effect of the influence radius on progress remained significant. In sum, we found that the effect of parameter *R* on progress was partially mediated by fragmentation and that the direct effect remained significant.

The angular noise *σ* had a strong effect on progress, as Fig. 7 illustrated. To test whether this effect is mediated by fragmentation, we focused on the simulation with a small influence radius (*R* = 0.03) as the effect of angular noise on progress was rather weak under *R* = 0.3. In addition, all runs quickly reached a consensus when the influence radius was large, leading to an extremely small variance in the degree of fragmentation when *R* = 0.3. For the simulations with the small influence radius, however, it turned out that 58.53 percent of total effect of angular noise on progress was mediated by fragmentation. The mediation was statistically significant according to the Preacher and Hayes test (coefficient = -.682, with a standard error of .319). What is more, Table 1 shows that the statistical effect of parameter *σ* turned insignificant in Step 3, showing that there is no support for a direct effect of parameter *σ* on progress.

**Table 1:** Mediation analysis results.

	Dependent variable		
	Avg. dist. from truth	Num. of clusters	Avg. dist. from truth
	(Step 1)	(Step 2)	(Step 3)
*Mediation analysis for influence radius *R**			
Influence radius *R*	**-0.054** (0.001)**	-6.108 (0.132)**	**-0.004** (0.001)**
Num. of Clusters			0.008 (0.000)**
Constant	0.080 (0.001)**	5.161 (0.077)**	0.037 (0.001)**
Explained variance	0.12	0.18	0.59
*Mediation analysis for angular noise *σ*, cases with *R* = 0.03*			
Angular noise *σ*	**-1.164** (0.163)**	-131.555 (2.032)**	**-0.483** (0.410)
Num. of Clusters			0.005 (0.003)
Constant	0.334 (0.010)**	16.624 (0.120)**	0.249 (0.048)**
Explained variance	0.32	0.79	0.34
*Mediation analysis for attraction to ground truth 1/*τ*, cases with *R* = 0.03*			
attraction by the ground truth 1/*τ*	**-2.868** (0.082)**	-51.819 (0.552)**	**-0.999** (0.112)**
Num. of Clusters			0.036 (0.002)**
Constant	2.047 (0.011)**	55.468 (0.073)**	0.046 (0.085)
Explained variance	0.12	0.49	0.17
*Mediation analysis for attraction to ground truth 1/*τ*, cases with *R* = 0.3*			
attraction by the ground truth 1/*τ*	**-0.660** (0.016)**	-58.646 (0.686)**	**-0.243** (0.020)**
Num. of Clusters			0.007 (0.000)**
Constant	0.472 (0.002)**	32.452 (0.091)**	0.241 (0.008)**
explained variance	0.17	0.45	0.25

* *p* < 0.05;

** *p* < 0.01

Also the strength *τ* of the attraction to the ground truth had a profound impact on progress, according to Experiment 1 (Fig. 8). The mediation analysis that focused only on those simulations where agents had a small influence radius of *R* = 0.03 demonstrated that 65.2 percent of this effect was mediated by fragmentation. Also this mediation was statistically significant (coefficient = -1.869, with a standard error of .080). Similar effects were found for the simulations with the bigger influence radius (*R* = 0.3). Here 63.1 percent of the total effect on progress was mediated by fragmentation. (Preacher and Hayes test: coefficient = -0.416, with a standard error of 0.016). Nevertheless, we found a direct effect of the strength *τ* of the attraction to the ground truth.

In sum, the influence radius *R* and the strength of attraction to ground truth *τ* were both responsible for a part of the relationship between progress and fragmentation. On the other hand, the effect of the parameter *σ* on progress was found completely mediated by fragmentation.

## Summary and Discussion

In this study, we asked why limited scientific progress and high degrees of disciplinary fragmentation tend to coincide. This correlation between fragmentation and progress could, in principle, result from three causal mechanisms (see Fig. 1). First, progress might decrease fragmentation. Second, fragmentation might limit progress and, third, there might be other variables that affect both outcomes and, thus, create a spurious correlation between them. Inspired by existing formal models and theories of how scientists’ generate insights, we focused on three independent variables that might affect scientists’ views and approaches: (i) the degree to which scientists are affected by the ground truth, (ii) the amount of social influence between fellow scientists, and (iii) the impact of noise on scientists views and approaches.

Our approach was purely theoretical. Based on existing philosophical theories and formal models of social influence dynamics, we developed a computational model of scientific disciplines consisting of a population of scientists who seek to identify the correct answer to a research question. By means of two simulation experiments and with the help of statistical methods, we were able to establish whether and under which conditions these assumptions about individual behavior generated fragmentation and progress and study why the two outcomes correlated.

**Fig 13:**
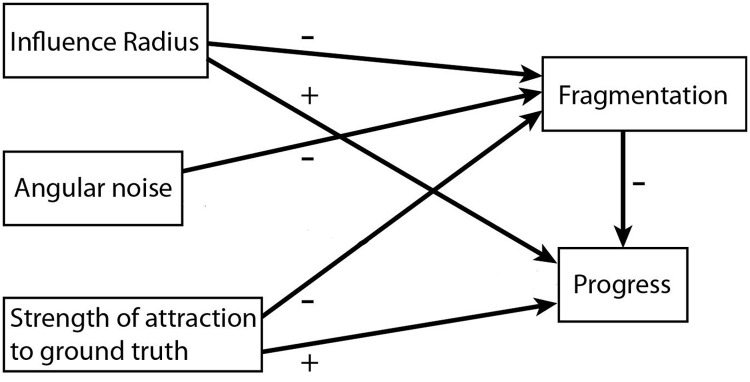
Effects that contribute to the correlation between fragmentation and progress. Each arrow represents a significant causal relationships among those that we hypothesized in Fig. 1, according to the mediation analysis on the results of our formal model.

Fig. 13 summarizes the main outcomes of our study, showing which of the possible mechanisms that can bring about the correlation between fragmentation and progress were supported by our simulation experiments and the statistical analyses. Each of the arrows represents an empirically testable hypothesis about the correlation between the respective variables.

We found that fragmentation hampered progress, but there was no effect in the opposite causal direction. In other words, limited progress is the consequence and not the cause of fragmentation. Thus, *the first answer to the question why progress and fragmentation correlate is that fragmentation causally affects progress*. This conclusion is consistent with philosopher T. Kuhn’s view of pre-paradigmatic science, where progress is limited because much intellectual energy is wasted arguing over fundamentals with rival schools, instead of developing an original research tradition [14].

In addition, *we also found that the two outcomes were influenced by the same variables, which also explains why fragmentation and progress correlate*. First, scientific progress and the degree of fragmentation turned out to depend on the strength of the attraction of the ground truth. Our model suggests that scientific disciplines in which views and approaches that are strongly influenced by the ground truth tend to experience less fragmentation and progress faster. The latter effect exists on top of the indirect effect via fragmentation.

Furthermore, it turned out that increasing the social influence radius decreases the amount of fragmentation and facilitates progress. Thus, this variable also explains why progress and fragmentation are correlated according to our model. An important finding of our study is that social influence facilitates progress. This contradicts the criticism often heard that social processes would distract scientists in their scientific exploration. However, being open to peer influence is not enough. Progress remains limited when agents fail to consider the position of other scientists with distant views, or when deviations from the main approach are not allowed. These variables, which were operationalized with the influence radius *R* and with angular noise *σ*, proved to have very strong effects.

In sum, *our model identifies two core mechanisms fostering scientific progress: (i) social interactions, and (ii) peer disagreement*. Similar conclusions have been reached also by contemporary philosophers of science, who argue that critical social interactions are the only way to acquire firm, and rationally based knowledge [36].

Our model is related to, but different from previous models of epistemic or fitness landscape exploration. In particular, our model assumes one single ground-truth in the epistemic space, instead of a rugged landscape [37]. Even though this simplification might appear incongruent with the constructivist ontology described by Feyerabend’s quote, we argue that this is actually not the case. Social constructivism, in fact, implies that knowledge constructed within each group is socially validated by the group itself. In a sense, it would require multiple ground truth to co-exist at the same time. This could, of course, be modeled by a rugged landscape where different groups of scientists stand on top of distinct local maxima without the immediate capacity to discern which group has reached the highest elevation. Remarkably, our simpler model can reproduce exactly the same distributional pattern of fragmentation without the need to assume the ruggedness of the fitness landscape. In fact, in our model, agents never know whether they have identified the ground-truth, but they keep exploring the two-dimensional space of possible answers until they reach a state where they are not changing their view anymore. In this sense, our model lacks an explicit notion of “epistemic significance”, e.g. an acknowledged evaluation of the explanatory power of a certain combination of approaches shared by all scientists belonging to the same field [38]. This implies that (i) there many approaches that can lead to the identification of the ground truth, and that (ii) agents in one cluster cannot immediately evaluate the views of agents into another distant cluster as less factual adequate than their own. The latter being a necessary condition for a representation of a constructivist ontology. Moreover, by not using a rugged landscape we avoided introducing further assumptions about the behavior of agents stuck in a local maximum.

Future modeling work is needed to assess the sensitivity of our results to changes in model assumptions and to derive further hypotheses about the conditions of progress and fragmentation in scientific disciplines. For instance, our model is build around the assumptions that there is one and only one answer to the research problem that the agents study, that agents’ initial views are uniformly (randomly) distributed around this point of truth, and that scientists are attracted by the ground truth with a force that scales linearly with distance. Studying how changing any of these assumptions affects model predictions is certainly a worthwhile line of investigation. Furthermore, future research could manipulate the total number of agents, and experiment with alternative assumptions about agents’ behavior when they reached the border of a bounded epistemic space. Finally, a further important extension will be to relax the assumption that all agents are equally affected by the ground truth, peer influence, and noise.

Obviously, a second crucial line of future research is to empirically test the predictions derived from our study. However, empirically studying which of the three causal mechanisms are responsible for the correlation between fragmentation and progress is very challenging, as the two outcomes and also the three independent variables are very difficult to quantify, and can hardly be manipulated experimentally. One promising approach to measure the degree of fragmentation in a discipline is to measure the degree of clustering in the network of citations in scientific publications [39]. Substantially more challenging, however, is measuring scientific progress, the degree to which a discipline has identified the true answers to their research questions. In fact, as the two quotes from the introduction indicate, the degree to which scientific insights are affected by signals from the ground truth is debatable, suggesting that it is often difficult to assess whether a discipline has identified the true answer to a given research question.

An additional challenge for empirical research is to test whether the three independent variables of our study are flexible—e.g. they might change over time—and whether they are in fact causally affected by the outcome variables. For instance, it appears possible that scientists who work in a highly fragmented field limit the degree to which they are influenced by disagreeing others, or even seek to increase differences to clusters of scientists who disagree [40]. In our simulation setup, we could exclude that agents change the degree to which they were influenced and that such effects altered our results. Future empirical studies, however, will have to statistically control for effects of the outcome variables on the independent variables. If empirical research provides support for these effects, it would also be interesting to explore the predictions of our model when such additional assumptions are included. For instance, previous modeling studies explored social influence dynamics when agents seek to intensify differences to others [41, 42]. Results indicate that such effects reinforce the formation and stability of clusters and can even give rise to polarized distributions of views in a discipline.

Our model presented a coherent theory of why fragmentation come about in scientific disciplines, and what consequences it has on collective progress. However, alternative explanations can be found in the literature. Many of these, in particular those developed by social scientists, point to the problematic behavior of researchers in the social sciences. For instance, in his critique of the field, sociologist S. Cole [5] has argued that empirical work “seems to have little impact on the beliefs of sociologists”(p. 15), that social scientists “select topics on non-theoretical grounds”(p. 14), that most “books are incredibly boring”(p. 28), or even that “sociologists just seem to enjoy stabbing each other in the back”(p. 29). Furthermore, the anthropological analysis of “scientific tribes” by Becher et al. describes sociologists to be very strict about methodological issues, to the point that the language of book reviews becomes overtly hostile [43, 44].

Another possible explanation might lie in the complexity of the subject of study. Positivist philosopher Auguste Comte (1798—1857) argued that knowledge develops faster the more general, the simpler, and the more independent is from other “departments”. Accordingly, he ranked the scientific disciplines established at his time in the following order: astronomy, physics, chemistry, biology, and sociology. Following Comte’s argument, it is possible to explain differences in levels of fragmentation across scientific disciplines as due to the different level of complexity of the subject of study. Comte’s hierarchy has been considered only as a theoretical entity for a long time, however, the recent abundance of bibliographic data has allowed researchers to actually put it under test. Landmark study by sociologist S. Cole could not find evidence in support of the existence of Comte’s hierarchy [45]. However, more recently, a substantial number of studies did found that scientific disciplines significantly differ from each other in a number of indicators—such as citation immediacy, article length, title length, shared references, use of graphs, anticipation frequency, average age at receiving Nobel prize, lecture disfluency, etc.—and that those differences are largely in line with the hypothetized hierarchy of sciences described by Comte [12, 46–50].

Our findings point to possible measures to foster scientific progress. First of all, the finding that fragmentation is the cause and not the consequence of progress suggests that decreasing fragmentation will have positive effects on scientific progress. What is more, our results also show how fragmentation can be reduced. First and foremost, the simulated disciplines progressed much faster when agents were strongly attracted by the ground truth, showing that progress is increased when views and approaches are frequently confronted with empirical data. It has been often criticized that many researchers tend to neglect the outcomes of empirical studies [5]. Our model supports that progress is undermined when the members of a discipline collectively disregard signals from the ground truth. Obviously, a common standard for interpreting such signals is needed, which is not yet the case in the social sciences. Second, increasing the radius of influence in the “interdisciplinary” consideration of distant views and different approaches can sensibly accelerate convergence. Third, noise had important effects on the model dynamics. It turned out that individual deviations from the behavior of immediate peers (social influence) and outcomes from empirical research (impact of ground truth) have desirable effects. Our results suggest that such deviations should, therefore, be supported (if deviations are idiosyncratic). To this regard, our results support the conclusion of Parker et al. [51], who studied the beneficial effects of deviations from the mainstream approach in peer review. A particularly interesting finding is that noise in the approaches of the agents (angular noise) decreases fragmentation even when agents have very a small influence radius. Thus, supporting deviations from the standard approaches in a field of subgroup might foster progress even under conditions where social influence is limited.

More concretely, our results could guide the design of social institutions that foster scientific progress. The most pervasive social institution in science is the peer-review system. Even though our model is highly abstract, it suggests that peer review could potentially foster progress, by exposing researchers to influence from others with different views and approaches. In reality, the complexity of the subject, the limited time and type of interaction, and the unbalanced incentives structure between authors and reviewers, are all factors that make peer review not well suited for long term discussions. In fact, as also suggested by philosopher of science Longino [36], we desperately need to create adequate *venues* for critical and interactive discussion to take place. This would be the best way to support scientific progress. Online technologies are already altering the way scientists do research, and they provide a good chance for fragmented fields to get out of their deadlocked situation. But there is still a long way to go towards a more participative, problem-oriented, and interdisciplinary science.

## Supporting Information

### S1 Model Dynamics Video

S1 Video Model Dynamics. The video illustrates the model dynamics of four typical simulation scenarios.
(MP4)

### S2 Supporting Information

S1_Text Supporting Information. Detailed description of computational experiments, mediation analysis, and boundary conditions.
(PDF)
