# Supplementary material for: On Disciplinary Fragmentation and Scientific Progress
Source: PLoS One. 2015 Mar 19;10(3):e0118747. doi: 10.1371/journal.pone.0118747 (PMC4366147; doi:10.1371/journal.pone.0118747)
Supplement: S1_Text — Detailed description of computational experiments, mediation analysis, and boundary conditions. (PDF) [file pone.0118747.s002.pdf]

Supporting Information for "On Fragmentation and Scientific Progress"

Stefano Balietti[1,*], Michael Mäs[1], Dirk Helbing[1]

1 Chair of Sociology, in particular Modeling and Simulation, ETH Zurich, Switzerland

∗ **E-mail: sbalietti@ethz.ch**

Simulation Experiments

Both simulation experiments were performed using MATLAB 7.14 on ETH Zurich's Brutus supercomputing cluster. In both simulation experiments, clusters of agents with similar views were identified with a standard hierarchical clustering algorithm [1,2] that identifies a set of agents as a cluster if their pairwise Euclidian distance is relatively small compared to the pairwise Euclidian distances to the members of other clusters. We chose a value of 0.1 as cutoff parameter of the hierarchical clustering algorithm.

Simulation Experiment 1

To study the effects of social influence, we manipulated two parameters. First, the interaction radius R was varied between 0.01 and 1 in steps of 0.01. Second, we studied the effect of the strength of social influence, manipulating parameter α between 0.01 and 0.99 in steps of 0.01. The effect of the ground truth on agents' approaches was manipulated using parameter τ, which we varied between values of 1 and 100 in steps of 1. Finally, we studied the effects of angular noise and position noise. The strength σ of the angular noise was varied between 0 and 0.1 in steps of 0.01 and the strength ϵ of the position noise was varied between 0 and 0.5 in steps of 0.1. Table 1 summarizes all parameter manipulations. In addition, the table reports the default value of each parameter. Unless mentioned explicitly, these default values were chosen for the analyses where one or more parameters were kept constant. For the interaction radius R, we studied two default values because we found that the system behaved in two fundamentally different ways depending on the value of R. In total, there were $100 * 99 * 100 * 11 * 6 = 6534 * 10^4$ combinations of parameter values that we studied in the experiment. We conducted 100 independent replications per parameter combination, arriving at a total of $6534 * 10^6$ simulation runs for Experiment 1.

We ran all simulations for 2,000 time steps, as it had turned out that by this moment the vast majority of runs had either reached a state of equilibrium or the dynamics had reached a state of temporal stability.

Table 1. Manipulations of model parameters in Simulation Experiment 1.

Parameter	Symbol	Default	Range (step)
Interaction Radius	R	0.03 ; 0.3	0.01 - 1 (0.01)
Strength of Social Influence	α	0.5	0.01 - 1 (0.01)
Strength of Attraction to Truth	τ	1	1 - 100 (1)
Angular Noise (Std.)	σ	0.01	0 - 0.05 (0.01)
Position Noise (Std.)	ϵ	0.1	0 - 0.5 (0.1)
Number of Simulation Steps	T	2000	1 - 20000
Number of agents	N	100	100
Length Velocity Vector at time 0	V_0	Unif(0,1) - 0.5	Unif(0,1) - 0.5

The latter was the case, for instance, in the ideal-typical run shown in Panel D of Fig. 2 of the main text, where the general social structure of the population had remained almost the same between the time steps 1,000 and 2,000. Obviously, when noise is included these temporarily stable distributions of views and approaches will change eventually, potentially leading to further scientific progress and changing degrees of fragmentation. However, as our aim was to study how fast disciplines progress and how much fragmentation they experience in this process, it was sufficient for us to focus our analyses on the first 2,000 time steps.

Simulation Experiment 2

We devised the following method to manipulate the initial degrees of fragmentation and progress. First, we randomly assigned 100 agents to c clusters of (about) the same size. In the experiment, we varied parameter c between 1 and 30 in steps of 1, in order to manipulate the degree of initial fragmentation. Next, the clusters were placed equally spaced on a circle with the ground truth in the center. To manipulate the initial degree of progress, we varied the radius of the circle, testing different initial distances from the ground truth. A problem with this approach is that the interaction radii of agents that belong to adjacent clusters on the circle can overlap, creating one big connected network component where all pairs of agents are directly or indirectly connected by influence relationships, rather than c unconnected clusters. Obviously, such as network cannot be considered fragmented. In order to avoid that our results are affected by this problem, we used two different intervals of initial distances from the ground truth (different radii of the circle) for those simulations where agents have a small influence radius R, and those simulation where R was large. The intervals were chosen such that the distance between the interaction radii were approximately the same for both conditions when the number of clusters was maximal ($c = 30$).

This led us analyze the interval (0.1 - 1) for $R = 0.03$, and (1.3892 - 2.2892) for $R = 0.3$. Both ranges were scanned in steps of 0.05 units. To be sure, also this method does not guarantee that clusters never formed a connected component. Whenever this was a problem for the interpretation of our results we, therefore, restricted our analyses to only those simulation runs where the number c of clusters was small enough to exclude that there was influence between clusters already at the outset.

To assure that our results were not affected by the choice of values for the remaining model parameters, we studied multiple parameter combinations. We studied two parameter values of the social influence radius ($R = (0.03, 0.03)$) and three values of the strength of social influence ($\alpha = (0.01, 0.5, 0.99)$). We kept constant the strength of the attraction to the ground truth ($\tau = 1$), and the levels of angular noise ($\sigma = 0.01$), and position noise ($\epsilon = 0.1$). We conducted 50 independent simulation runs per parameter combination, leading to a total $50 * 30 * 19 * 3 * 2 * 2 = 342,000$ simulation runs for Experiment 2.

Mediation Analyses

As we know from Experiment 2, fragmentation affects progress, but there is no effect in the opposite direction. This means that we can conduct Baron and Kenny's mediation test [3] on the data of Experiment 1 for each of the parameters R, σ, and τ that significantly affected both progress and fragmentation.

Using the same operationalization as in Experiment 1, we measured progress as agents' average absolute Euclidian distance from the ground truth at time step 2,000. Also the degree of fragmentation of the disciplines was operationalized in the same way as in Experiment 1. However, we calculated fragmentation already after half of the simulation runs was completed (at 1,000 time steps), to measure the degree of fragmentation that the simulated disciplines experienced in the process of making scientific progress. We replicated the mediation analyses also with fragmentation measured at $t = 2,000$ and found virtually identical effects. To simplify the display of the statistical results, we included in the regressions reported in the Mediation Analysis table in the main text only those simulations that varied the respective parameter (R, σ, or τ) and kept all remaining parameters at their default values (see Table 1). Whenever we found that results differed for other values of the remaining parameters, we conducted additional analyses, as documented in the main text.

A Note on Boundary Conditions

In our model, we do not assume that the epistemic space is bounded, i.e. the space that agents can explore is infinite. Obviously, it is a deep philosophical question whether or not this is a correct model and we are unable to contribute to this debate here. However, we decided to not implement boundaries, because this would have forced us to include further potentially problematic assumptions, as detailed below.

Assuming bounds implies that one has include additional assumptions about agents' behavior when they reach such bounds. One option would be to assume so-called "periodic boundary conditions", which implements that agents that reach one border of the epistemic space reappear at the opposite border and then continue exploring with their previous approach. However, in the context of our study we deemed it very implausible that scientists who have adopted an extreme view will suddenly adopt opposite views. Alternatively, one could assume that agents who have reached the boundary simply stop exploring. This assumption, however, leads to the formation of fragmented clusters at the border of the epistemic space. However, fragmentation is one of the core outcome variable of our study and we sought to avoid making assumptions that might affect our results. A third option would be to assume that agents are reflected by the boundaries. This implements that agents that reached the border of the epistemic space adopt the opposite approach and start exploring the space in the opposite direction. We tested this option, but it turned out that it also fostered the formation of clusters close to the boundaries. This effect occurs when groups of agents collectively reach a border. Under these conditions, some group members might be reflected by the border and move in the opposite direction earlier than others. However, it is likely that these updated agents are still within the interaction radius of those group members whose approach is still pointing towards the border. When social influence takes places between agents with opposite approaches it leads to a marked decrease in the exploration speed of all agents involved. Therefore, reflecting boundary conditions favor the formation of new clusters and the merging of existing ones close to the boundaries. If the attraction to the ground-truth is weak, this creates an "herding at the boundaries" effect. Even though this is very interesting, it does not seem to mirror any known real-world behavior of scientists, and we considered it an artifact of the boundary assumptions.

In a nutshell, we deemed these options problematic and, therefore, decided to implement no boundaries. Nevertheless, this discussion shows that more empirical research on scientists strategies and exploration methods is needed, in order to develop more valid formal models.

References

1. Everitt B (1974) Cluster Analysis. Heinemann, London.

2. Hubert L, Köhn HF, Steinley D (2009) The Sage Handbook of Quantitative Methods in Psychology, Thousand Oaks: Sage Publications, chapter Cluster Analysis: a Toolbox for MATLAB. pp. 444–511.

3. Baron R, Kenny D (1986) The moderator–mediator variable distinction in social psychological research: Conceptual, strategic, and statistical considerations. Journal of Personality and Social Psychology 51: 1173.
